# Supplementary material for: Coexistence of resistance oscillations and the anomalous metal phase in a lithium intercalated TiSe2 superconductor
Source: Nat Commun. 2021 Sep 9;12:5342. doi: 10.1038/s41467-021-25671-8 (PMC8429654; doi:10.1038/s41467-021-25671-8)
Supplement: Supplementary file 1 — Supplementary Information [file 41467_2021_25671_MOESM1_ESM.pdf]

Supplementary information to:

Coexistence of resistance oscillations and the anomalous metal phase in a lithium intercalated $TiSe_2$ superconductor

Menghan Liao, [1] Heng Wang,[1] Yuying Zhu,[1,2] Runan Shang,[2] Mohsin Rafique,[1] Lexian Yang,[1,3] Hao Zhang,[1,2,3] Ding Zhang,[1,2,3,4] * and Qi-Kun Xue [1,2,3,5] *

[1]*State Key Laboratory of Low Dimensional Quantum Physics and Department of Physics, Tsinghua University, Beijing 100084, China*

[2]*Beijing Academy of Quantum Information Sciences, Beijing 100193, China*

[3]*Frontier Science Center for Quantum Information, Beijing, 100084, China*

[4]*RIKEN Center for Emergent Matter Science (CEMS), Wako, Saitama 351-0198, Japan*

[5]*Southern University of Science and Technology, Shenzhen 518055, China*

*Email: dingzhang@mail.tsinghua.edu.cn, qkxue@mail.tsinghua.edu.cn

Contents

Supplementary Note 1. Lithium intercalation (Supplementary Fig. 1-2)

Supplementary Note 2. Anisotropy of the upper critical field (Supplementary Fig. 3)

Supplementary Note 3. Evaluation of physical parameters from transport measurements (Supplementary Fig. 4-7)

3.1 Carrier density

3.2 Mobility

3.3 Electron mean free path

3.4 Superconducting coherence length

3.5 Lithium content

Supplementary Note 4. Influence of radio frequency filters on the transport results (Supplementary Fig. 8-11)

Supplementary Note 5. Extraction of the CDW temperature (Supplementary Fig. 12)

Supplementary Note 6. Additional data on the resistance oscillations (Supplementary Fig. 13-17)

Supplementary Note 7. Magneto-resistance in the anomalous metal phase (Supplementary Fig. 18)

Supplementary Note 8. Hall coefficients as a function of doping at 1.6 K and 230 K (Supplementary Fig. 19)

References 1-7

Supplementary Note 1. Lithium intercalation

We perform time-of-flight secondary ion mass spectroscopy (TOF-SIMS) to demonstrate the presence of lithium ions in $TiSe_2$ after gating. Supplementary Fig. 1 compares the signals from two flakes on the same substrate. The $TiSe_2$ flake that is not contacted by the electrodes (Supplementary Fig. 1a) serves as the reference sample. For the sample grounded by the electrodes (inset of Supplementary Fig. 1b), gating at 6 V, 300 K for 30 min drives lithium ions into the material. Supplementary Fig. 1b shows that the signal of $^{6}Li^{+}$ in the electrically grounded sample is over one order of magnitude higher than that of the reference sample (Supplementary Fig. 1a). Furthermore, we obtain $^{6}Li^{+}$ signal right at the surface of the intercalated flake, indicating that lithium ions can easily migrate up to the topmost layer. We note that the rapid decay of $^{6}Li^{+}$ signal in the SIC substrate is probably due to the repulsion of positive ions by the sputtered O_2^{+} ions, as was previously reported [1].

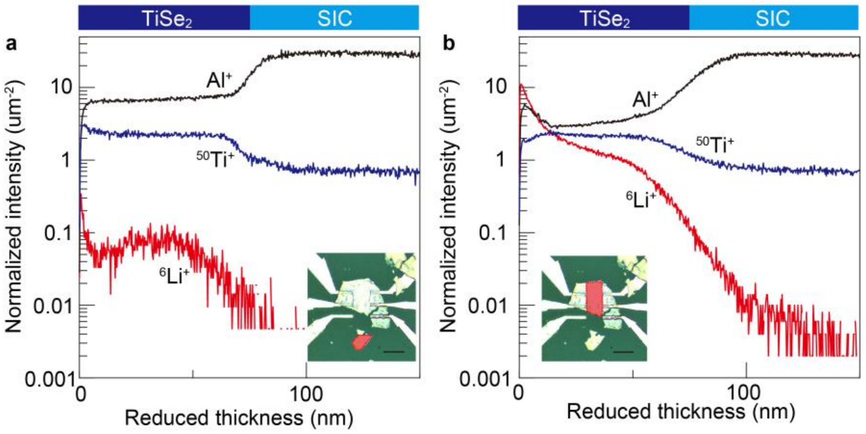

Supplementary Figure 1 Depth profiles of the Al^{+}, $^{50}Ti^{+}$ and $^{6}Li^{+}$ intensities from the top surface of $TiSe_2$ to the substrate (SIC) by TOF-SIMS. Signals are obtained from the ungated (**a**) and the gated (**b**) $TiSe_2$ flakes, respectively. The sudden increase of Al^{+} signal at the reduced thickness of about 80 nm marks the boundary of $TiSe_2$ and SIC, since Al^{+} should be present only in SIC. Insets of panel **a** and **b** show the optical images of the device (scale bar: 30 μm), in which the shaded regions demarcate the areas where the signals are extracted. No lithium ions intercalate into the flake that is not electrically contacted (highlighted in the inset of

panel **a**), such that it serves as a reference. The other flake with the central area highlighted by red, as shown in the inset of panel **b**, was gated at 6 V, 300 K for 30 min before loading into the analysis chamber.

To further elucidate the intercalation process, we develop *in-situ* atomic force microscopy that allows us to scan the topography of our sample during gating. This technique benefits from the fact that the electrochemical process occurs via a solid-state substrate such that the sample surface is accessible by scanning probes. Supplementary Fig. 2a shows the measurement scheme, which combines AFM with electrical measurements. We keep scanning a small area of 10×0.625 μm^2 (time cost: 16 s, resolution: 256×32, indicated in the inset of Supplementary Fig. 2b) during the gating process. Simultaneously, we record the sample resistance. Supplementary Fig. 2b shows how the height and the resistance changes as we ramp up the gate voltage (top panel of Supplementary Fig. 2b) with time. The resistance shows a sudden drop at about $t = 25$ min, which correlates with the rapid increase of height. It indicates that lithium ions start to intercalate into the sample. Interestingly, after this event, the height stays almost constant while the resistance keeps dropping. To better correlate the electrical property with the topography, we plot in Supplementary Fig. 2c the sample height as a function of the conductance ($1/R$). The height of the sample increases from 80 nm to a plateau of around 90 nm while the conductance gets a fourfold increase from 0.05 S to 0.2 S. We speculate that as long as certain amount of lithium ions intercalates into the sample, the interlamellar gaps become expanded. Further intercalation only accumulates more lithium ions in those gaps without changing the lattice. The 12.5% expansion is also in agreement with the previous study on a similar system (TiS_2) [2]. Starting from about $t = 45$ min, extended gating causes another jump in height from 90 nm to around 100 nm, probably due to the formation of a second layer of lithium ions in the interlamellar gaps. For samples S1 and S2 discussed in the main text, the conductance at room temperature only increases by a factor of 3.5, as indicated in the bottom panel of Supplementary Fig. 2c. Therefore, there exists only one layer of lithium ions in their interlamellar gaps.

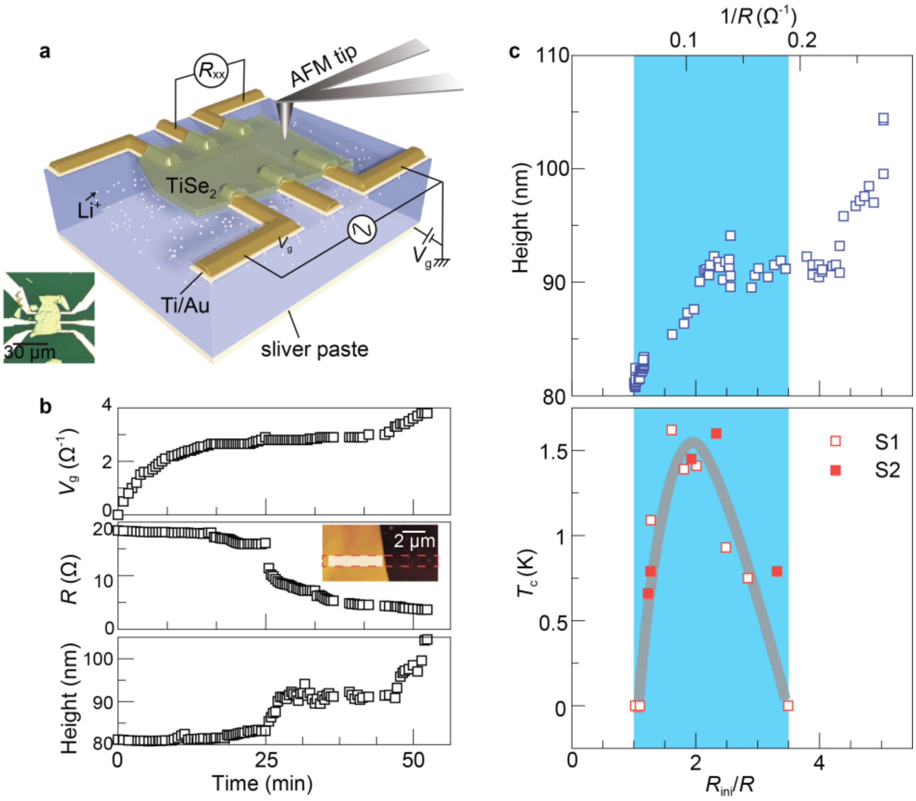

Supplementary Figure 2 Thickness variation of $TiSe_2$ upon lithium intercalation measured by *in-situ* atomic force microscopy. a, schematic illustration of the *in-situ* AFM measurements. Lower left panel shows the optical image of the device. **b,** time dependence of the applied gate voltage (V_g), the sample resistance (R) and the height of the flake. Inset shows one AFM image. The dashed rectangle marks the region scanned during gating. The white rectangle indicates the region over which the average height is extracted. **c** (top): height of the $TiSe_2$ flake upon lithium intercalation as a function of conductance ($1/R$). (bottom): superconducting transition temperature of samples S1 and S2 as a function of their normal state conductance (at room temperature). Here the bottom axis is the normalized conductance by dividing the conductance at each gated stage over the initial conductance. Blue shades indicate the region of the superconducting dome.

Supplementary Note 2. Anisotropy of the upper critical field

We carried out the angular dependent magnetic field study of the superconducting state in a dilution refrigerator equipped with a vector magnet (5-2-2 T). During the cooling of the dilution refrigerator from 300 K to about 230 K, we juggled between applying the gate voltage on (3 V) and turning it off. This procedure assures that the sample resistance stays at a value that is half of the initial one in the pristine sample. This gating procedure results in a Li_xTiSe_2 sample in the underdoped regime. At the base temperature (20 mK), we ramped up the magnetic field along different directions such that the angle (θ) between the magnetic field and the sample plane rotated from -10 to 90 degrees.

Supplementary Fig. 3 plots the sample resistance at different magnetic fields and θ. We obtain $B_{c2,0^\circ} = 0.085$ T and $B_{c2,90^\circ} = 0.94$ T at 20 mK. We use these critical field values to calculate the coherence length and the superconducting thickness based on the 2D Ginzburg-Landau formula and obtain ξ_{GL}= 62 nm, $d_{sc} =$ 20 nm. The coherence length estimated here is also consistent with the value obtained for sample S2 in the underdoped regime (Fig. 2d).

Despite that fact that this sample has a thickness of 80 nm, the angular dependent upper critical field still follows the 2D Tinkham model such that $(B_{c2}(\theta)\sin(\theta)\,/B_{c2,0^\circ})^2 + |B_{c2}(\theta)\cos(\theta)/B_{c2,90^\circ}| = 1$ [3]. In comparison, the upper critical field of a 3D superconductor with strong anisotropy should follow the 3D Ginzburg-Landau formula: $B_{c2}(\theta) = B_{c2,0^\circ}/\left[\sin^2\theta + \left(\frac{B_{c2,0^\circ}\cos(\theta)}{B_{c2,90^\circ}}\right)^2\right]^{1/2}$ [3]. It indicates that Li_xTiSe_2 behaves as a stack of 2D superconducting layers, probably due to the increased spacing between the $TiSe_2$ layers after lithium intercalation.

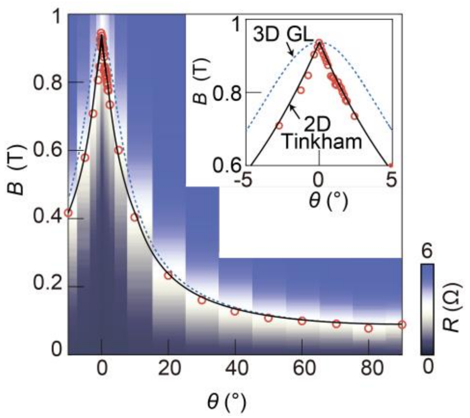

Supplementary Figure 3 Color coded sample resistance at the base temperature of a dilution refrigerator (20 mK) as a function of magnetic field and the angle. Here the angle is between the applied field and the *a*-*b* plane of the $TiSe_2$ crystal. Dark blue region indicates the superconducting state while the light blue region corresponds to the normal state. White color corresponds to half of the normal state resistance. Solid and dotted curves are fits by considering the 2D Tinkham model (solid) and the 3D anisotropic Ginzburg-Landau model (dotted), respectively.

Supplementary Note 3. Estimation of parameters of $TiSe_2$ from transport measurements

3.1 Carrier density

Supplementary Fig. 4 plots the optical images of sample S1-S3 and their corresponding Hall resistances measured at 1.6 K at different gating stages. All Hall resistance curves exhibit linear behaviors, reflecting the single band nature of $TiSe_2$ at low temperatures. We note that a recent quantum oscillation study also demonstrates that there exists only a single elliptic electron pocket at low temperatures [4], consistent with the band structure revealed by angular resolved photoemission spectroscopy [5]. Therefore, despite the possible involvement of both electron and hole transport in $TiSe_2$ at room temperature [4], its low temperature electrical property originates from a single electron band. We extract the areal density (n_T) and the 3D carrier density (n_{3D}) by the formulas:

$$n_T = \frac{1}{R_H e}, \tag{1}$$

$$n_{3D} = \frac{1}{R_H e d}. \tag{2}$$

Here d is the sample thickness. R_H is the Hall coefficient and is determined from the slope of the Hall resistance curves.

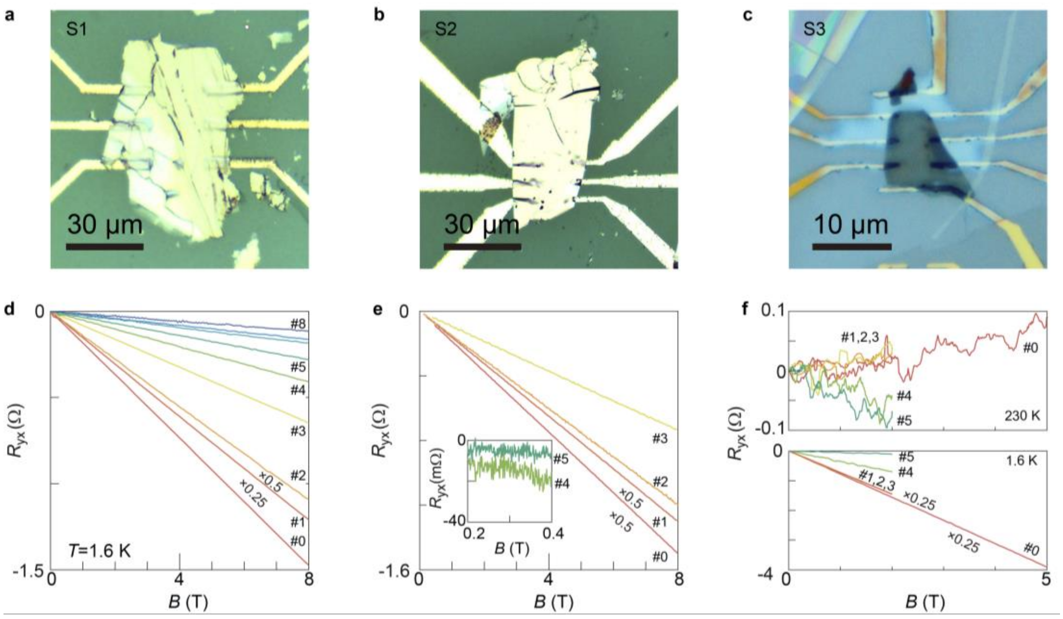

Supplementary Figure 4 a-c, Optical images of samples S1, S2 and S3. Sample S1 and S2 are about 50 nm thick, sample S3 is 10 nm thick. The thicknesses were measured by atomic force microscope. To protect the thinner sample from degradation, sample S3 was covered by an extra layer of hexagonal boron nitride (h-BN). **d-f,** Hall resistances of the three samples at

different gating stages. Data in **d**, **e** and the lower panel of **f** were obtained at 1.6 K. Data in the upper panel of **f** were obtained at 230 K.

3.2 Mobility

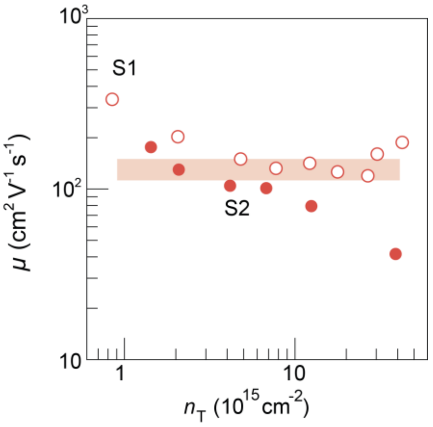

Supplementary Figure 5 Mobility of samples S1 and S2 as a function of total carrier densities.

By using the Hall density and the sheet resistance (R_S) of the sample at 1.6 K, we evaluate the mobility following:

$$\mu = \frac{1}{R_S n_T e}. \tag{3}$$

Supplementary Fig. 5 shows the extracted μ as a function of n_T. Our samples have mobility about 100 cm^2/V·s across a wide doping range. The intercalated lithium ions seem to contribute mostly to the increasing charge carriers. They do not cause a dramatic increase in scattering. This dichotomy again echoes with our conclusion from AFM study (section 1) that lithium ions are in the interlamellar gaps. Effectively, the "charge reservoir" layer of lithium ions are separated from the conducting layer of $TiSe_2$.

3.3 Electron mean free path

To further evaluate the mean free path l, we start with the definition:

$$l = v_F \tau, \tag{4}$$

where v_F is the Fermi velocity and τ is the relaxation time. The Fermi velocity is related to the Fermi momentum k_F by $v_F = \frac{\hbar k_F}{m^*}$, where m^* is the effective mass. For a single electron band with the valley degeneracy of g and spin degeneracy of 2, we obtain:

$$k_F = \left(2\pi \frac{n_{2D}}{g}\right)^{1/2} \quad \text{for a 2D electronic system,} \tag{5}$$

and $$k_{F,ab} = \frac{1}{\eta}\left(3\pi^2 \frac{n_{3D}}{g}\right)^{1/3} \quad \text{for a 3D anisotropic electronic system.} \tag{6}$$

Here η^3 is the anisotropic factor such that $k_{F,ab}\eta^3 = k_{F,c}$, where $k_{F,ab}$ and $k_{F,c}$ are the Fermi momentum in the *a*-*b* plane and the *c*-direction, respectively. We obtain the expression of the Fermi velocity as:

$$v_F = \frac{\hbar}{m^*}\left(2\pi \frac{n_{2D}}{g}\right)^{1/2} \quad \text{(2D),} \tag{7}$$

$$v_{F,ab} = \frac{\hbar}{m^*_{ab}}\frac{1}{\eta}\left(3\pi^2 \frac{n_{3D}}{g}\right)^{1/3} \quad \text{(anisotropic 3D).} \tag{8}$$

The other parameter in Eq. (4) to determine l is the relaxation time. From the Drude model, τ is related to the conductivity by:

$$\tau = \frac{m^*\sigma}{ne^2}, \tag{9}$$

Here either the 2D or 3D conductivity of σ can be used depending on whether a 2D or 3D carrier density of n is inserted. By combining Eq. (7)/(8) with Eq. (9), we obtain:

$$l = \frac{\hbar\sigma_{2D}}{n_{2D}e^2}\left(2\pi \frac{n_{2D}}{g}\right)^{1/2} \quad \text{(2D),} \tag{10}$$

$$l = \frac{\hbar\sigma_{3D,ab}}{n_{3D}e^2}\frac{1}{\eta}\left(3\pi^2 \frac{n_{3D}}{g}\right)^{1/3} \quad \text{(anisotropic 3D).} \tag{11}$$

Based on the results in section 1 and 2, we argue that Li_xTiSe_2 consists of a stack of $TiSe_2$

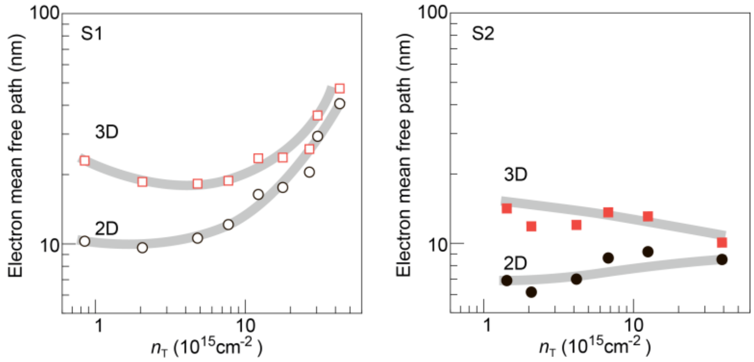

layers separated by lithium ions. We therefore use Eq.(10) to evaluate the mean free path of each conducting layer. Here, $\sigma_{2D} = \frac{1}{NR_s}$ and $n_{2D} = \frac{n_T}{N}$, where N is the total number of $TiSe_2$ layers. We use $g = 3$ by taking into account the specific band structure of $TiSe_2$—a single electron pocket with three-fold valley degeneracy [4,5]. The mean free path l extracted by this method is plotted in Fig. 2d of the main text. For comparison, we also use Eq. (11) to estimate the mean free path. η is determined from ref. [4]. Supplementary Fig. 6 compare the mean free paths estimated from Eq. (10) and Eq. (11). In general, they are on the same order of magnitude and suggest that $l < \xi$, thus the Li_xTiSe_2 superconductor is in the dirty limit.

Supplementary Figure 6 Electron mean free path of samples S1 and S2 estimated by considering Li_xTiSe_2 as either a stack of 2D electronic system or an anisotropic 3D system

3.4 Superconducting coherence length

Supplementary Fig. 7 shows a typical superconducting transition of Li_xTiSe_2 and its magnetic field response. We extract the temperature dependence of the upper critical fields (B_{c2}) in Supplementary Fig. Sb and estimate the in-plane superconducting coherence length by using the slope dB_{c2}/dT close to $T_{c,0}$: $\xi_{ab} = \sqrt{\frac{\Phi_0/2\pi}{-dB_{c2}/dT \cdot T_{c,0}}}$, where Φ_0 is the flux quantum.

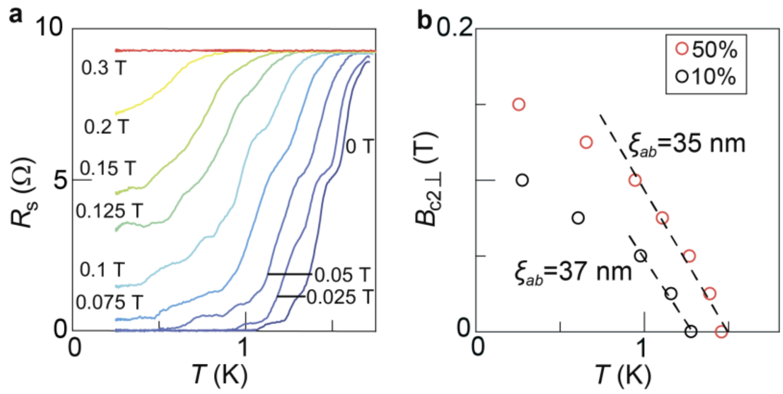

Figure 7 a, Temperature-dependent resistance at different magnetic fields for sample S2 (50 nm thick) near optimal doping. **b**, Perpendicular upper critical field as a function of temperature for sample S2. Red and black circles are data points extracted by defining the transition temperature as the point where the resistance drops to 10% and 50% of normal state resistance (R_n), respectively. Dashed lines are linear fits to determine the slopes close to T_{c0}.

3.5 Lithium content

Here we estimate the lithium content for sample S2 at nearly optimal doping, i.e. $n_T(\#4) = 6.8 \times 10^{15}$ cm^{-2}. The initial carrier density of pristine $TiSe_2$ with the same thickness of 50 nm is 0.85×10^{15} cm^{-2}, as we determine from sample S1. Therefore, the increase of carrier density is about 6×10^{15} cm^{-2}, or 7.2×10^{13} cm^{-2} per $TiSe_2$ layer. By using the in-plane lattice constant of $TiSe_2$: $a_{TiSe_2} = 3.54$ Å, we estimate that each unit cell gains 0.08 electron from the lithium ions. If we use 0.85 as the effective charge transfer per lithium atom—the value reported in C_6LiC_6 [6], the lithium content can be 0.094. Recently, it was reported that in bilayer graphene with superdense lithium, the effective charge transfer per lithium atom is 0.33 [6]. If we use this ratio, we estimate that the lithium content is 0.24.

One immediate question is whether the periodic structure that generates the resistance oscillations is caused by the intercalated lithium ions. In bilayer graphene, a superdense ordering of lithium with a lattice constant of a_{Li}=3.1 Å was observed [6]. A less dense packing of lithium may result in a larger a_{Li} that is closer to the lattice constant of $TiSe_2$. Consequently, one may expect a Moiré pattern with a period on the 100 nm scale. However,

the lithium content in the optimally doped situation is: x=0.1-0.24, which is one order of magnitude lower than that required for a dense packing. Furthermore, it is unlikely that lithium ions stay mobile at around 1 K, at which we observe clear evolution of the periodic structure (Fig. 3d, e, g in the main text).

Supplementary Note 4. Influence of radio frequency filters on the transport results

We compare the temperature dependent resistance curves obtained with and without the LC filters. Supplementary Fig. 8 characterizes the transmission of the wire with and without the filter. We furthermore show data obtained with different excitation current in Supplementary Fig. 9. The resistance curves overlap when the excitation current is below 1.5 μA. To optimize the signal to noise ratio, we chose 1 μA for the measurements at other magnetic fields and doping levels. Supplementary Fig. 10 compares the data obtained at two doping levels with and without filters. In the optimal doped sample (Supplementary Fig. 10a,b), the resistance saturates at higher values at the same magnetic field if filters are removed. In the overdoped sample (Supplementary Fig. 10c,d), however, the resistance does not show saturation down to lowest temperature of 250 mK even if there are no filters. We show in Supplementary Fig. 11 the data obtained from the underdoped sample without filters. There too the resistance does not show saturation in the measured temperature range.

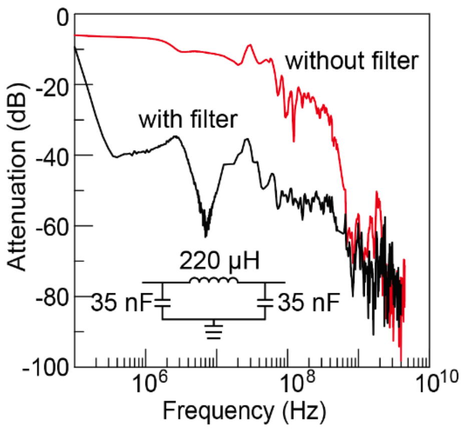

Supplementary Figure 8 Attenuation of the measurement line. The transmission was measured between the BNC connector at the breakout box and the pin on the sample holder of the ^{3}He insert. We used a network analyzer (Keysight E5063A) with a frequency range of 100 kHz to 4.5 GHz. The inset illustrates the π-filter we employed in the breakout box.

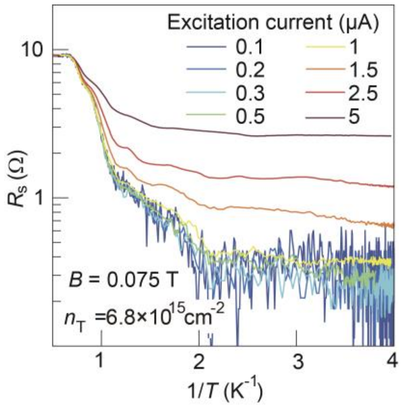

Supplementary Figure 9 Comparison of data retrieved with different excitation currents. These data were obtained with the filters installed.

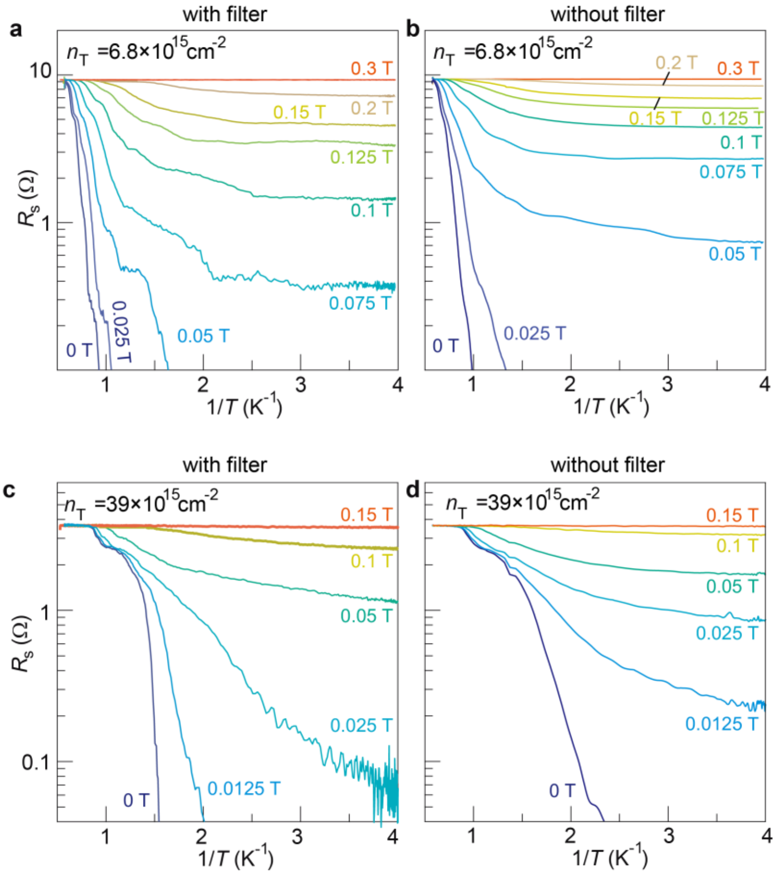

Supplementary Figure 10 Comparison of the data obtained with and without the application of filters. **a**,**c**, Arrhenius plot of the data at two different doping levels. The same data are presented in Fig. 3. **b**,**d**, Arrhenius plot of the data obtained at the two doping levels with the same sets of magnetic fields but without the installation of the filters. In the overdoped situation of **d**, even removing the filters cannot induce a resistance plateau at low temperatures, suggesting the missing of the anomalous metal phase.

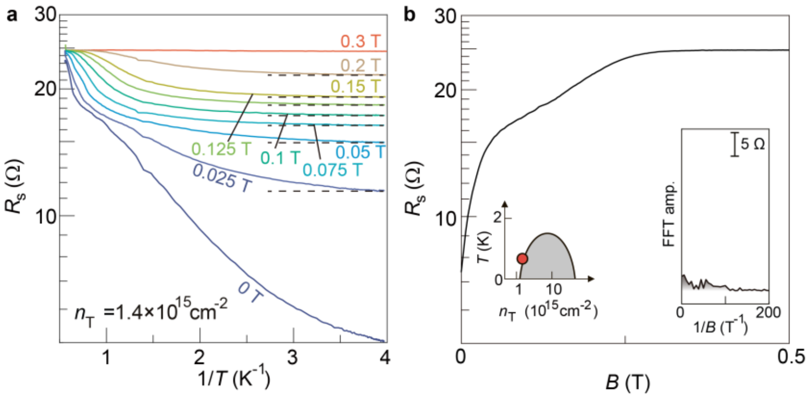

Supplementary Figure 11 **a**, Arrhenius plots of the sheet resistances at selected magnetic fields for the sample in the underdoped regime. This data set was obtained without installing the filters. Although the resistance curves show departures from a thermally activated behavior at low temperatures, none of the curves show a plateau in the measured temperature range. **b**, Magnetoresistance at 0.25 K of the sample in the underdoped regime. Left inset to **b** indicates the doping level in the schematic phase diagram. Right inset is the Fast-Fourier Transforms (FFT) of the magnetoresistance curve.

Supplementary Note 5. Extraction of the CDW temperature

We extract the CDW temperature T_{CDW} from the derivative of $R_s(T)$. To avoid uncertainty in determining the local minimum brought by the noise in the derivative curve, we carry out the following procedure: (1) linearly fit the data in the two temperature sections: 180 to 195 K and 195 to 210 K. (2) extrapolate the two linear fits and use the crossing point as the position where the local minimum/kink occurs. Supplementary Fig. 12 a-c show exemplary curves at three different doping levels for sample S1. Supplementary Fig. 12d summarizes T_{CDW} at different carrier densities. They stay almost constant over the entire doping range. The decrease of symbol size represents the drop of the magnitude for the resistance peak (Fig. 1c, d of the main text).

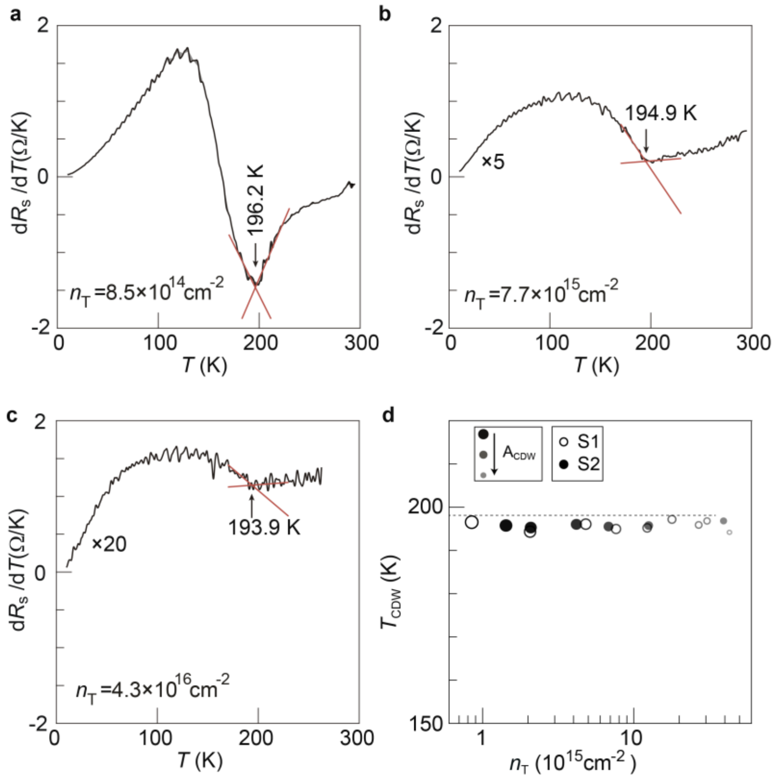

Supplementary Figure 12 a-c Exemplary derivative of temperature dependent resistance from sample S1. The carrier densities of the three cases are in the underdoped (**a**), nearly optimal doped (**b**), to the over-doped regime (**c**). The red lines are linear fits of data in the temperature ranges of [180, 195] K and [195, 210] K, respectively. **d** Extracted CDW temperature as a function of total carrier densities for samples S1 and S2. The decreasing size of the circles reflect the smaller magnitude of the resistance peak at higher doping (defined in the inset of Fig. 1d).

Supplementary Note 6. Additional data on the resistance oscillations

Supplementary Fig. 13 shows the magnetoresistance curves obtained at the same doping and temperature but with two different sweep rates of the magnetic field. The resistance spikes occur at the same positions irrespective of the sweeping rate. Supplementary Fig. 14 shows the angular dependent study of the resistance oscillations. The results indicate that the oscillations occur at the same perpendicular magnetic field with different in-plane magnetic fields. Supplementary Fig. 15 shows the magnetoresistance oscillations for sample S2 in the underdoped situation. Supplementary Fig. 16 gives an example of the smoothed background and trace after subtracting this background. The smoothed background is obtained by running a moving average over data points within 9.2 mT. Supplementary Fig. S17 shows the phase diagram of the anomalou metal region at a higher doping level than that shown in Fig. 4d. Here, this region also overlaps with the region showing resistance oscillations.

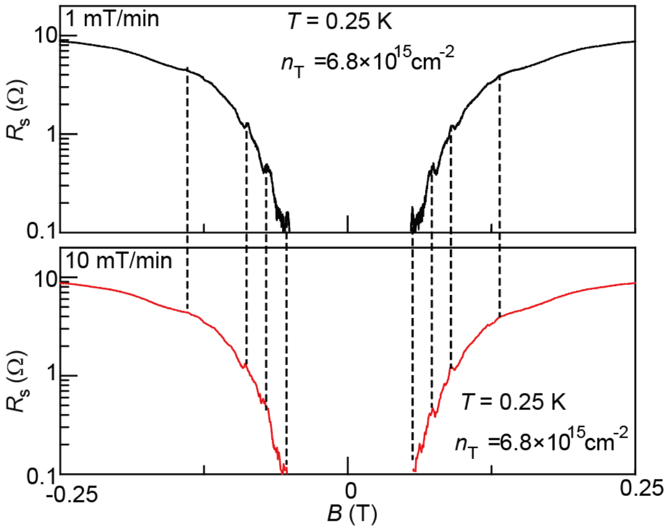

Supplementary Figure 13 Magneto-resistance acquired with two different sweeping rates. The vertical dashed lines mark the spikes in the magneto-resistance, which occur at the same magnetic fields irrespective of the sweeping rate.

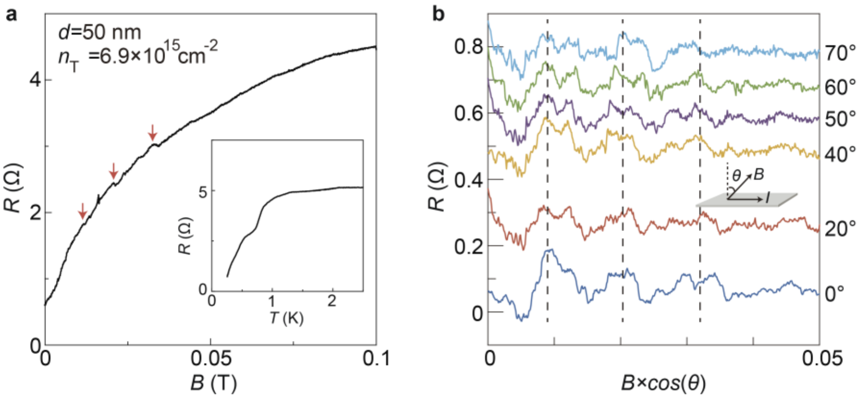

Supplementary Figure 14 Angular dependent study of the resistance oscillations. **a**, Magneto-resistance at 0.25 K for a 50 nm thick sample near the optimal doping. Here the magnetic field is applied perpendicular to the sample plane. Red arrows mark the peak positions of the resistance oscillations. Inset shows the temperature dependent resistance at zero magnetic field. **b**, The resistance oscillations as a function of the perpendicular magnetic field. Each curve is obtained by subtracting a smoothed background. We rotate the sample in the magnet. The inset illustrates the direction of the magnetic field relative to the sample plane.

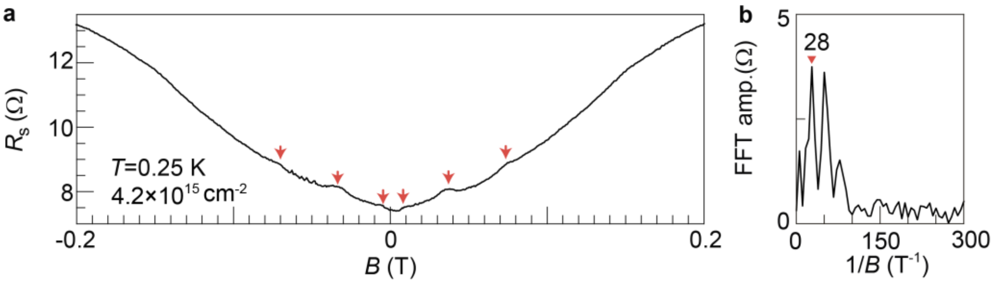

Supplementary Figure S15 Magneto-resistance oscillations of sample S2 in the underdoped regime. Arrows in panel a mark the resistance spikes. Arrow in panel b indicates the peak position in the FFT that we use to calculate the domain size.

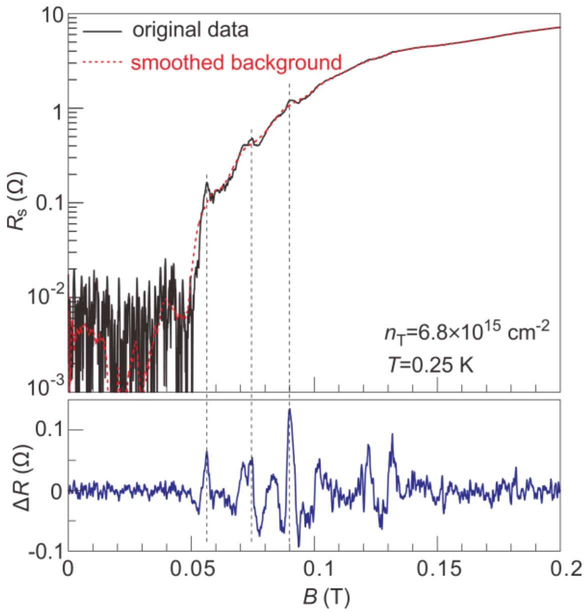

Supplementary Figure 16 Top panel: experimentally measured magnetoresistance (solid) and its smoothed background (dotted). Bottom panel shows the curve after subtracting the smoothed background. Vertical dashed lines mark the three pronounced spikes.

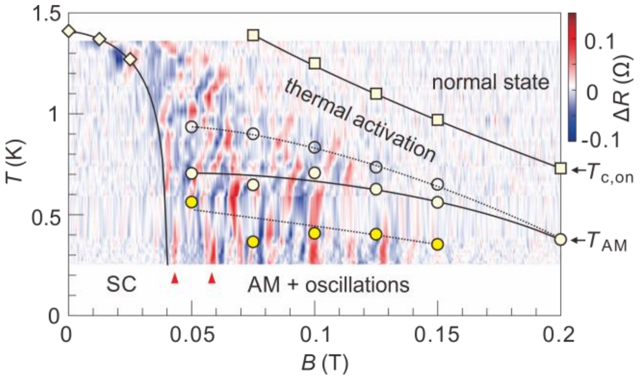

Supplementary Figure 17 Phase diagram and resistance oscillations obtained after subtracting a smoothed background at $n_T = 13 \times 10^{15}$ cm^{-2}. The empty circles demarcate the temperatures at which the temperature dependent resistance deviates from a thermally activated behavior, as defined by empty circles in the middle panel of Fig. 4a. The other two types of circles represent T_{AM} and T_{AM0}, as explained in the main text. The squares/diamonds are the temperature points where the resistance drops to 90% or 0.4% of the normal state resistance, respectively. They represent the onset and the full entrance of superconductivity, respectively. The solid curves are guide to the eye. Red triangles mark the corresponding peak positions in Fig. 3b.

Supplementary Note 7. Magneto-resistance in the anomalous metal phase

One promising theoretical model considers the tunneling of vortices across the superconducting puddles as the mechanism for the dissipation in the anomalous metal phase [7]. In this scenario, the magneto-resistance is expected to follow:

$$R_s = R_Q \, exp\left[C \frac{\pi}{2} \frac{\hbar/e^2}{R_n} \left(\frac{B-B_{c2}}{B_{c2}}\right)\right], \tag{12}$$

where $R_Q = h/4e^2$ and C is a dimensionless constant of order unity. Because our system behaves as a stack of 2D superconducting layers, we modify the right hand side of the equation by further dividing R_Q and $\hbar/e^2$ by N—the number of $TiSe_2$ layers:

$$R_s = \frac{R_Q}{N} exp\left[C \frac{\pi}{2} \frac{\hbar/e^2}{NR_n} \left(\frac{B-B_{c2}}{B_{c2}}\right)\right]. \tag{13}$$

We regroup the terms and assign two dimensionless quantities: $G = ln\left(NR_s/R_Q\right)$ and $\beta = (B - B_{c2})/B_{c2}$. Apparently, G is directly proportional to β such that:

$$G = C \frac{\pi}{2} \frac{\hbar/e^2}{NR_n} \beta. \tag{14}$$

Supplementary Fig. 18 plots in the main panels G as a function of β for the data obtained at two doping levels. In the highlighted region, the magneto-resistance curves at different temperatures overlap. It corresponds to a resistance plateau as function of temperature at a fixed magnetic field—key characteristic of the anomalous metal phase. In this specific region, the data indeed follows the simple linear dependence of Eq. (14) with zero intercept. The deviation from the linear behavior in the highlighted region is caused by the resistance oscillations. For the two doping levels, we obtain $C =$1.32 and 0.82 respectively, in agreement with the theory in ref. [7].

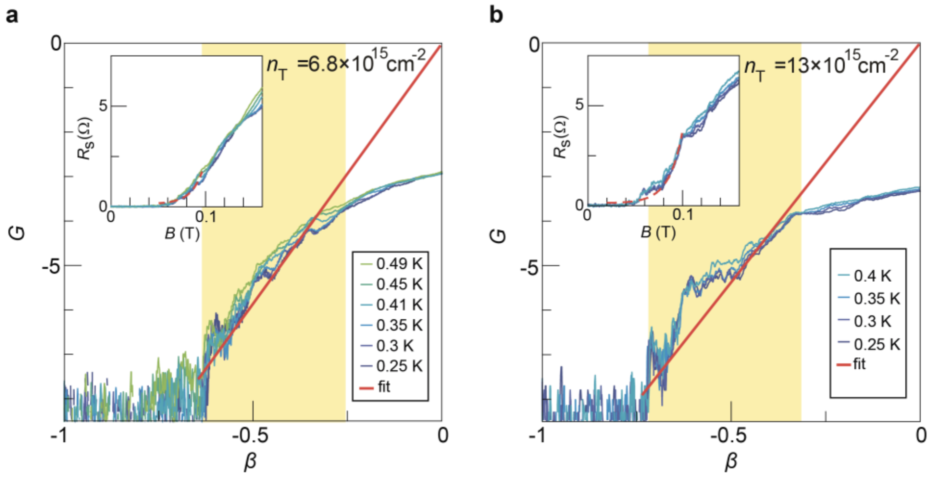

Supplementary Figure S18 Magnetoresistance of sample S2 at low temperatures for two doping levels. The yellow rectangles highlight the anomalous metal regime. The red lines are theoretical fits by using Eq. (14) to the anomalous metal regime. Insets show the magneto-resistance data together with the theoretical curves by putting the extracted C into Eq. (13).

Supplementary Note 8. Hall coefficients as a function of doping at 1.6 K and 230 K

For pristine $TiSe_2$, it hosts both electron and hole carriers at room temperature. The transport is dominated by electron carriers at temperatures below the CDW transition (around 200 K). We measure on sample S3 the Hall coefficients at 230 K (above the CDW transition) and 1.6 K as a function of lithium intercalation. Supplementary Fig. 19a shows the temperature dependent resistance curves. These data sets are also used in the main text to calculate the area of the resistance peak as a function of doping. The Hall data for sample S3 are already presented in Supplementary Fig. 4f. Supplementary Fig. 19b compares the Hall coefficients extracted from sample S3 at 1.6 K and 230 K. Notably, for gating stages 1 to 3, the Hall coefficients are positive at 230 K—indicating a dominant hole transport—and transit to negative values at 1.6 K. At gating stage 4 and 5, the sample enters the overdoped regime and the Hall coefficients are negative at both 230 K and 1.6 K.

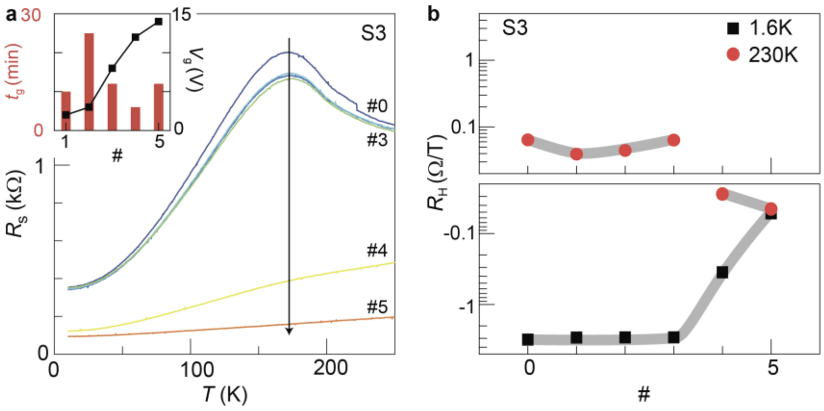

Supplementary Figure 19 a, temperature dependent resistance of sample S3 at different gating stages (indicated in the inset). **b,** Hall coefficients of sample S3 measured at 1.6 K and 230 K as a function of gating stages. In the over-doped regime (gating stages 4 and 5), the Hall coefficients stay negative at both 1.6 K and 230 K. It indicates that the Fermi level shifts upward substantially and the electronic condition for CDW—coexistence of electrons and holes—is no longer satisfied.

References:

[1] Tellez, H., *et al*. New perspectives in the surface analysis of energy materials by combined time-of-flight secondary ion mass spectrometry (ToF-SIMS) and high sensitivity low-energy ion scattering (HS-LEIS) *J. Anal. At. Spectrom.* **29**, 1361 (2014).

[2] Whittingham, M. S. Electrical Energy Storage and Intercalation Chemistry. *Science* **192**, 1126 (1976)

[3] Saito, Y., *et al.* Metallic ground state in an ion-gated two-dimensional superconductor. *Science* **350**, 409-413 (2015).

[4] Knowles, P., *et al*. Fermi surface reconstruction and electron dynamics at the charge-density-wave transition in $TiSe_2$. *Phys. Rev. Lett.* **124**, 167602 (2020).

[5] Chen, P., *et al*. Charge density wave transition in single-layer titanium diselenide. *Nat. Commun.* **6**. 8943 (2015).

[6] Kühne, M., *et al*. Reversible superdense ordering of lithium between two graphene sheets. *Nature*, **564**, 234-239 (2018).

[7] Shimshoni, E., Auerbach, A., and Kapitulnik, A., Transport through quantum melts, *Phys. Rev. Lett.* **80,** 3352 (1998).
